# Discovering the Recondite Secondary Metabolome Spectrum of *Salinispora* Species: A Study of Inter-Species Diversity

**DOI:** 10.1371/journal.pone.0091488

**Published:** 2014-03-12

**Authors:** Utpal Bose, Amitha K. Hewavitharana, Miranda E. Vidgen, Yi Kai Ng, P. Nicholas Shaw, John A. Fuerst, Mark P. Hodson

**Affiliations:** 1 School of Pharmacy, The University of Queensland, Brisbane, Queensland, Australia; 2 School of Chemistry and Molecular Biosciences, The University of Queensland, Brisbane, Queensland, Australia; 3 Metabolomics Australia, Australian Institute for Bioengineering and Nanotechnology, The University of Queensland, Brisbane, Queensland, Australia; California Department of Public Health, United States of America

## Abstract

Patterns of inter-species secondary metabolite production by bacteria can provide valuable information relating to species ecology and evolution. The complex nature of this chemical diversity has previously been probed via directed analyses of a small number of compounds, identified through targeted assays rather than more comprehensive biochemical profiling approaches such as metabolomics. Insights into ecological and evolutionary relationships within bacterial genera can be derived through comparative analysis of broader secondary metabolite patterns, and this can also eventually assist biodiscovery search strategies for new natural products. Here, we investigated the species-level chemical diversity of the two marine actinobacterial species *Salinispora arenicola* and *Salinispora pacifica*, isolated from sponges distributed across the Great Barrier Reef (GBR), via their secondary metabolite profiles using LC-MS-based metabolomics. The chemical profiles of these two species were obtained by UHPLC-QToF-MS based metabolic profiling. The resultant data were interrogated using multivariate data analysis methods to compare their (bio)chemical profiles. We found a high level of inter-species diversity in strains from these two bacterial species. We also found rifamycins and saliniketals were produced exclusively by *S. arenicola* species, as the main secondary metabolites differentiating the two species. Furthermore, the discovery of 57 candidate compounds greatly increases the small number of secondary metabolites previously known to be produced by these species. In addition, we report the production of rifamycin O and W, a key group of ansamycin compounds, in *S. arenicola* for the first time. Species of the marine actinobacteria harbour a much wider spectrum of secondary metabolites than suspected, and this knowledge may prove a rich field for biodiscovery as well as a database for understanding relationships between speciation, evolution and chemical ecology.

## Introduction

Marine bacteria from different phylogenetic groups produce secondary metabolites that play multiple ecological functions within their marine chemical environment [1]. Such secondary metabolites are produced under the pressure of natural selection and may act as agents of interaction (e.g. antagonism or competition) with other microorganisms in a community or as signals for communication within populations of the same species [2], [3]. The production of antibiotics by microbes is a typical example of such a response and is primarily thought to be a defence against microbial competitors; however, these molecules have also been found to have roles as quorum-sensing signals, or other functions that help stabilize microbial communities [3]. Thus, fine scale comparative analysis of secondary metabolite production at the species level provides a means by which to explore and understand species biology, ecology and evolution. In addition, it is one of the keys to understanding the chemical diversity underlying biodiscovery of novel previously unknown pharmaceuticals and other chemical products. Nonetheless, to date limited attention has been paid to such fine-scale comparative analysis of natural product diversity in bacterial species, especially concerning marine bacteria.

The genus *Salinispora* was the first obligate marine actinobacterial genus to be described and members are widely distributed in tropical and sub-tropical marine sediment and marine sponges to depths of 2000 m [4], [5]. The genus comprises three closely related species *S. tropica*, *S. arenicola* and *S. pacifica* [6], [7]. Like their actinobacterial terrestrial counterparts, *Salinispora* produce numerous secondary metabolites with diverse possible pharmaceutical applications. The compound salinosporamide A, isolated from *S. tropica* is currently in Phase 1 clinical trials in patients with multiple myeloma, lymphomas, leukaemia and solid tumours [8]. This genus has also been found to produce secondary metabolites with diverse activities, for instance arenimycin, rifamycins, staurosporine, saliniketal A and B, cyclomarazines and cyclomarins, as well as hydroxamic acid siderophores [4], [8], [9], [10].

Bacterial species classified based on their 16S rRNA gene sequences can vary greatly at the genomic level [11]. These genomic differences are mainly found in isolated islands – regions of the chromosome which are known to contain genes associated with ecological adaptation [11]. To date, most of these reported *Salinispora* species-derived compounds are polyketides or non-ribosomal peptides, or hybrids thereof, and their biosynthesis is accomplished by large multi-enzyme complexes, the polyketide synthases (PKS) or the non-ribosomal peptide synthases (NRPS) [12], [13]. Analyses of the *S. tropica* and *S. arenicola* genomes have revealed the presence of putative natural product biosynthesis genes, comprised of PKS and NRPS, with a large percentage of the genome (8.8% and 10.9% respectively) devoted to secondary metabolite biosynthesis, which is greater than the percentage of such genes in the *Streptomyces* secondary metabolite genome sequence [12]. In 2007, the *S. arenicola* CNS-205 genome sequencing project revealed a 5.8 Mbp genome (CP00850) with at least 30 distinct metabolite gene clusters [12]. These results also suggest that the production of secondary metabolites may be linked to ecological niche adaptation within this group of bacteria, and that the acquisition of natural product biosynthetic genes represents a previously unrecognized influence driving bacterial diversification [14]. Taken together, these findings suggest that species of the genus *Salinispora* possess the capacity to produce a large number of secondary metabolites. Only a limited number of the potentially wide spectrum of such metabolites have actually been detected to date (e.g. represented by the salinosporamides, saliniketals, sporolides, arenimycins, cyclomarins, etc. listed above).

In addition to the production of diverse secondary metabolites, this genus has attracted major interest for the novel phenomenon of species-specific production of such secondary metabolites [15]. Jensen and co-workers (2007) have previously shown that *Salinispora* was the first bacterial genus to be identified as having species-specific secondary metabolite production correlated to their phylogenetic diversity at the species level. Core compounds have been produced by a specific species, for example the compounds salinosporamide A-J, sporolide A and B, and an antiprotealide were only found to be produced by *S. tropica* [14]. However, recent studies have shown that staurosporine, which was previously isolated from *S. arenicola* [16] is also produced by *S. pacifica* [14]. The vertical gene transfer of the *sta*D gene sequences between two sister taxa *S. arenicola* and *S. pacifica*, are responsible for the production of staurosporine in *S. pacifica* [14].

Horizontal gene transfer (HGT), the exchange and stable integration of genetic material from different strains and species, is a major evolutionary force [17]. The process of genetic exchange allows bacterial species to acquire traits from distantly related organisms and as a consequence aids adaptation to the changing environment [18]. Recent research has highlighted that HGT may have occurred throughout a major part of the bacterial genome [12], [15]. Genes acquired by HGT play multiple roles: virulence, metabolism, resistance to antibiotics and the long-term maintenance of organelles [19]. Although it is clear that a large part of the *Salinispora* genome acquired gene clusters for secondary metabolite production via HGT, the ecological and evolutionary significance of these mechanisms remain unclear.

Evidence of HGT in *Salinispora* species comes from a phylogenetic study of the PKS genes associated with the rifamycin biosynthetic gene cluster (*rif*) in *S. arenicola* and *Amycolatopsis mediterranei*, the original source of this compound [20], [21]. Rifamycins are naphthalenic ansamycin antibiotics produced by a number of soil- and marine-derived actinomycetes, for example *Amycolatopsis mediterranei* [22]. These compounds elicit their antibacterial activity through the specific inhibition of RNA synthesis via binding to the beta sub-unit of RNA polymerase [23]. Semi-synthetic rifamycin derivatives, for instance rifampicin, have been used as antibiotic therapies against *Mycobacterium tuberculosis* and *Mycobacterium leprae*, the causative agents for tuberculosis and leprosy, respectively. The naturally occurring variant rifamycin B is the parent molecule for other biologically active rifamycin compounds and rifamycin B is further processed either by natural enzymatic modification or by semi-synthetic mechanisms to produce the biologically active rifamycin analogues O, SV and S [24].

Numerous methods have been used to screen bacterial secondary metabolites for useful biopharmaceuticals [25]. One such method is bioactivity-guided screening, which allows detection of compounds with specific biological activity. For example, salinosporamide A was primarily isolated through bioactivity guided assays [8]. However, this method is clearly biased to specific targets in the activity assays, as well as the repeated “discovery” of known compounds and is limited by the availability of suitable assay methods. An alternative method to identify secondary metabolites is via chemical screening, which can identify the presence of diverse compound classes in a complex set of samples. Traditional screening methods involve separation and isolation of compounds followed by their identification but such methods may require time-consuming optimization of separation conditions for each compound and elaborate methods for identification. Chemical screening is also constrained by marked differences in the chromatographic and spectroscopic properties of the natural compounds. For example, UV/visible light detection by diode array detector-coupled liquid-chromatography is widely used but has limitations for the detection of certain compound classes, may lack specificity and spectra are sparsely represented (and therefore difficult to search) in structural databases [26]. Some other methods offer novel and innovative approaches for screening and/or identifying secondary metabolites, such as phylogenetic analysis at the genomic level [27], genome screening approaches (e.g. PCR-based identification of coding sequences [28] and functional genome analysis [29]. However, these approaches are often limited by the need for conservation of sequence with known genes (e.g. for design of PCR primers) and still require chemical analysis and identification of downstream products arising from the gene expression. Therefore, new approaches are needed to identify chemical signals produced by these microorganisms for selection of potentially useful compound libraries and to better understand microbial chemical ecology and evolution. For example, as it is now clear that variation in metabolite production exists between the two *Salinispora* species [15], more comprehensive analytical and data-mining techniques are needed to explore the secondary metabolite profiles of this genus.

The genome speaks of what compounds could potentially be produced as it codes for the machinery to make production possible. However, it is the phenotypic secondary metabolome that tells the story of which metabolites are actually available to the organism in its chemical arsenal for interacting with its external microbial community and habitat. Metabolomics is the comprehensive analysis of the biochemical content of cells, tissues or bio-fluids, usually from analysis of extracts [30]. Typically metabolomics experiments have utilised NMR- and/or MS-based analytical techniques to explore the metabolite content of experimental samples. Liquid Chromatography-Quadrupole Time of Flight-Mass Spectrometry (LC-QToF-MS) has received much attention in recent years for microbial metabolic fingerprinting studies as well as in many other fields of biology [25], [31], [32]. In this study, high resolution UHPLC-QToF-MS combined with multivariate/chemometric approaches were applied to investigate the secondary metabolome of *S. arenicola* and *S. pacifica* in order to elucidate the differences between their secondary metabolite profiles and to distinguish these two taxonomically identical species. Here we have highlighted a number of secondary metabolites that are responsible for differentiating two species at the metabolic and taxonomic level. Furthermore, from these data we confirm the first evidence of rifamycin O and W production in *S. arenicola*.

## Materials and Methods

### Sample preparation

**Figure 1:**
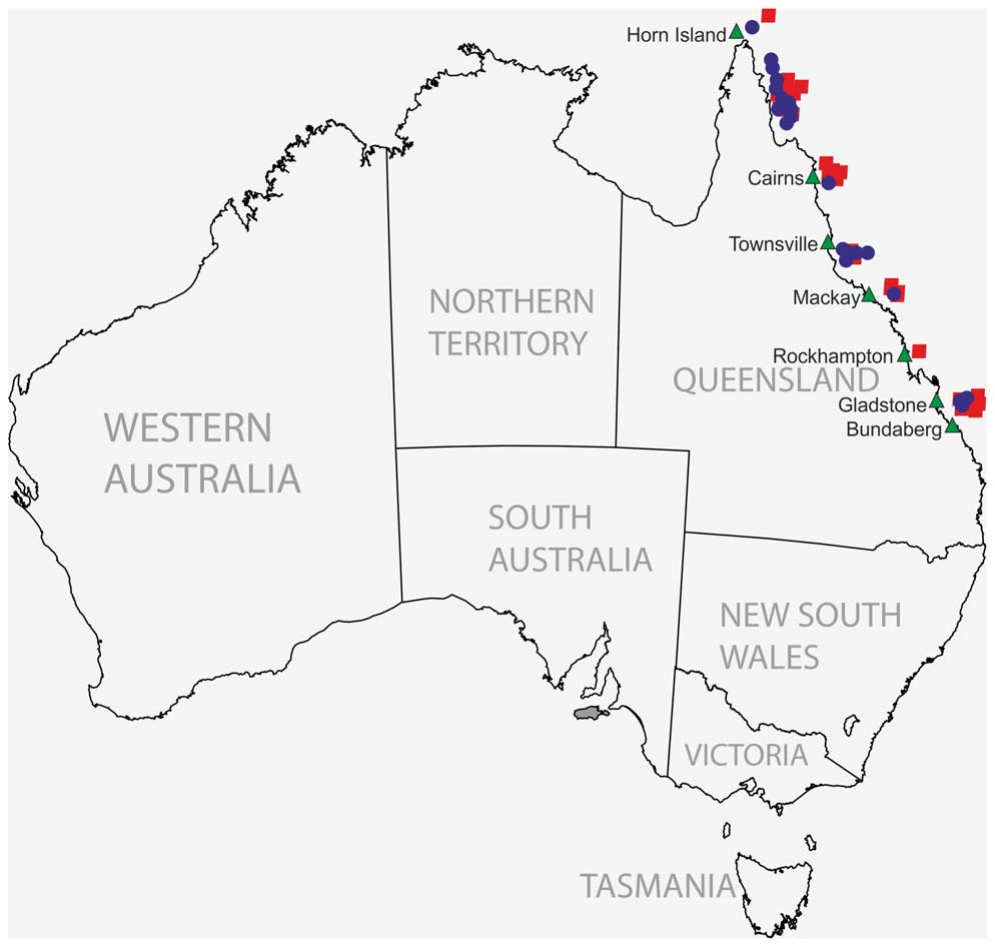
Distribution of sampling sites from which the 46 *S. arenicola* (blue) and *S. pacifica* (red) strains were collected. Bacterial species were spread over both Northern and Southern regions of the Great Barrier Reef (GBR) (over ∼2500 km).

This investigation involved the growth and analysis of 46 strains of *Salinispora* from two different species to obtain the secondary metabolite profiles. *Salinispora* isolates were collected at various locations along the Great Barrier Reef (GBR), Queensland, Australia, an area frequently studied for its tropical marine ecosystem (Figure 1). The isolation and taxonomic identification of *Salinispora* isolates has been previously reported [9], [33]. Strains used for rifamycin W identification were reported by Ng and co-workers [34]. In the present study the isolates were cultivated on Difco Marine Agar 2216 for 56 days at 28°C [33], until black pigmentation of the colonies was established in all strains. Between two and five biological replicates were grown for each of the 46 strains. The mycelial cell mass was harvested by scraping it off the growth medium using a scalpel blade and pooled in a pre-weighed 1.5 mL centrifuge tube. The net mass for each sample was recorded and subsequently used to normalise the data obtained from the extracts, based on the precise biomass extracted per sample. The same procedure was repeated using a blank agar plate to obtain a blank extract. To extract the secondary metabolites from the cell mass 1 mL of ethyl acetate was added to the sample and tube, and shaken for 90 minutes at room temperature. The tube was positioned vertically and the layers of ethyl acetate and *Salinispora* extract were allowed to separate before the ethyl acetate layer from each tube was transferred by pipette into three clean 1.5 mL centrifuge tubes. The ethyl acetate was then removed (by evaporation) from the extract using a vacuum centrifuge (Savant Instruments, Hicksville, NY) for 1 hour. The dried extract was resuspended at 15% of the original volume by adding 30 μL methanol and then 120 μL of MilliQ (Millipore, Bedford, MA, USA) water to produce a 20∶80 methanol:water solution. The extract solution was stored at −80°C until use. Before HPLC-MS analysis the samples were thawed and filtered using a sterile, 4 mm diameter, 0.2 μm PTFE membrane syringe filter (Phenomenex, Sydney, Australia). After filtration, the samples were kept at ∼4°C prior to injection. The injection volume was 20 μL.

#### UHPLC-QToF-MS analysis

The chromatographic separation of compounds in *Salinispora* extracts was performed using Ultra High Performance Liquid Chromatography (UHPLC) on an Agilent 1290 series system (Agilent Technologies, USA). The UHPLC was coupled to an Agilent 6520 high resolution accurate mass (HRAM) QToF mass spectrometer equipped with a multimode source (Agilent) and controlled using MassHunter acquisition software, (B. 02.01 SP3 - Agilent). Separation was achieved using a 2.1×50 mm, 1.8 μm ZORBAX SB C18 column (Agilent). The chromatographic analysis was performed using 5 mM aqueous ammonium acetate (mobile phase A) and acetonitrile (mobile phase B) at a flow rate of 0.15 mL/min. The column was pre-equilibrated for 15 minutes with 80% (v/v) A and 20% (v/v) B. After injection, the composition of mobile phase was changed from 20% (v/v) B to 100% (v/v) B over a period of 50 min, composition held at 100% (v/v) B for 5 min, and then returned to the starting composition of 20% (v/v) B over next 5 min. The column was re-equilibrated using 20% (v/v) B for 15 min prior to the next injection. The total chromatographic run time was 75 min.

A dual nebulizer electrospray source was used for continuous introduction of reference ions. In MS mode the instrument scanned from *m/z* 100 to 1000 for all samples at a scan rate of 0.8 cycles/second. This mass range enabled the inclusion of two reference compounds, a lock mass solution including purine (C_5_H_4_N_4_ at *m/z* 121.050873, 10 μmol L^−1^) and hexakis (1H, 1H, 3H-tetrafluropentoxy)-phosphazene (C_18_H_18_O_6_N_3_P_3_F_24_ at *m/z* 922.009798, 2 μmol L^−1^). Multimode (i.e. simultaneous Electrospray Ionisation [ESI] and Atmospheric Pressure Chemical Ionization [APCI]) with Fast Polarity Switching (FPS) was employed to ionise compounds after chromatographic separation. The QToF-MS conditions used for these experiments are given in Table S1. Methods for the identification of both rifamycin O and W are detailed in Text S1.

### Data analysis and Molecular Formula Generation

Data analysis was performed using Agilent MassHunter Qualitative software (Version B.05.00). The Molecular Feature Extractor (MFE) algorithm within MassHunter Qualitative analysis software was used to extract chemically qualified molecular features from the LC-QToF-MS data files. For empirical formula generation, the Molecular Formula Generator (MFG) algorithm was used. This algorithm uses a wide range of MS information, for instance accurate mass measurements, adduct formation, multimer formation and isotope patterns to generate a list of candidate compounds. The maximum elemental composition C_60_H_120_O_30_N_30_S_5_Cl_3_Br_3_ was used to generate formulae. MFG can automatically eliminate unlikely candidate compounds and rank the putative molecular formulae according to their mass deviation, isotopic pattern accuracy and elemental composition.

#### Chemometric analyses

The LC-MS molecular feature-extracted datasets were further processed using Agilent Mass Profiler Professional (MPP) software (Agilent) to extract and align peaks/features from the chromatograms of all extracts of the two *Salinispora* species (observations), resulting in a total of 3341 putative metabolite features (variables). To aid the data-mining process, the LC-QToF-MS data file of the blank sample was also analysed to extract features and to use as a background reference. These reference ions were removed from all samples in the matrix.

The resulting data matrix (144 observations; 3341 variables) was then exported as .csv and imported to SIMCA-P (version 13.0.3.0; MKS Umetrics AB, Umea, Sweden). All data were log transformed, mean centred and scaled to unit variance prior to multivariate analysis. Unsupervised analysis using Principal Component Analysis (PCA) was initially performed to reveal any outliers resulting from technical/instrumental/processing procedures and to assess any groupings or trends in the dataset. Thereafter supervised analysis was performed, where appropriate, whereby predetermined groupings were used to classify the data, using Orthogonal Projection to Latent Structures-Discriminant Analysis (OPLS-DA). The scores and loadings plots from these analyses, which describe the multivariate relationships of the observations and variables respectively, along with other metrics such as S-plots and VIP lists, were then used to determine the features (metabolites) that contribute to the differences between the experimental groups (*Salinispora* species). The selected features were used to generate putative molecular formulae in order to search for and identify compounds via database query, MS/MS fragmentation patterns and comparison to authentic standard(s).

## Results

### Global metabolic profiling with High Resolution Accurate Mass-Mass Spectrometry (HRAM-MS)

In order to extract the maximum number of secondary metabolites, a non-targeted extraction method was used. Ethyl acetate is a medium polarity solvent that is widely used and is capable of extracting a large percentage of compounds other than those that are extremely polar or non-polar. As an organic solvent, ethyl acetate has the additional benefit of denaturing and thus causing precipitation of macromolecules such as proteins, as well as causing the partition of highly polar analytes to the aqueous layer. Although untargeted, the choice of solvent should dictate that resultant extracts contain much of the secondary metabolome of interest.

A neutral mobile phase pH was used to be applicable to both positive and/or negative ionisation, with the use of fast polarity switching to detect sequentially both positive and negative ions. In addition, a multimode ion source was employed to maximise the ionisation of compounds that preferentially ionise in one source only. Although these strategies undoubtedly reduce the sensitivity of detection for specific ions, the methodology was employed as an initial “one shot” approach to enable the rapid screening of extracts.

### Inter-species chemical diversity

**Figure 2:**
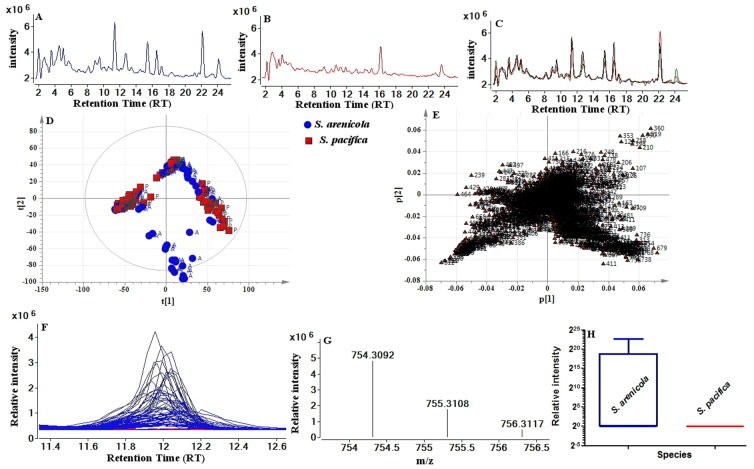
Identification of species-specific compounds in extracts of *S. arenicola* and *S. pacifica* by UHPLC-QToF-MS and PCA. (A) Total ion current (TIC) chromatograms of *S. arenicola* and (B) *S. pacifica* (C) TICs from the same (*S. arenicola*) subset of samples (three replicates) to highlight the reproducibility of acquisition. For ease of interpretation, chromatograms of 2 to 24 minutes are shown to illustrate the period of chromatography during which most compounds elute. Full chromatograms for both species are available in the Supplementary information (Figure S6). (D) Principal component analysis (PCA) scores plot, PC1 (t[1]) versus PC2 (t[2]) showing the variation in the profiles of secondary metabolites from two species: *S. arenicola* (A, blue) and *S. pacifica* (P, red). Each symbol represents one bacterial strain described by all detected metabolites. (E) Inspection of the 2-D loadings plot for PC1 vs. PC2 reveals the variables responsible for the spatial arrangement of samples. (F) Extracted ion chromatogram (EIC) of *m/z* 754.3092 from both species, showing clear species differences in the abundance of this metabolite *S. arenicola* (blue) and *S. pacifica* (red). (G) MS spectrum for EIC peak. (H) Box-and-whisker plot of the abundance of the 754 ion in the two species (P<.0001).

The LC-MS chromatograms obtained from two species were compared visually by using Total Ion Current chromatograms (TICs) in the mass range of 100 to 1000 *m/z* at a retention time window 2–50 minutes in order to detect differences between secondary metabolite production profiles. At the population level, the average number of compounds varied from ∼300 in *S. arenicola* to ∼150 in *S. pacifica* samples. Figure 2 (A, B) shows typical LC-MS profiles for the two species, *S. arenicola* and *S. pacifica*, and reveals differences between these two species. Figure 2C shows the metabolic profile of three biological replicates obtained from each sample obtained from a single strain. The consistency of these results for all the samples highlights the reproducibility of the biological replicates.

Initially, unsupervised analysis by PCA was used to identify any outliers and assess any groupings or trends in the data set. Results obtained from the PCA scores plot (illustrating the relative similarities or differences of the sample extracts of the two species) in Figure 2D shows that no clearly defined separation exists between the two bacterial species based upon the major sources of variation within their (bio)chemical profiles. The scores plot also confirms that no technical outliers are present but that some biological “outliers” are apparent, such as the sub-cluster of samples from *S. arenicola*. The variables responsible for any groupings or clusters in the data can be determined from the loadings plot Figure 2E; as an example, the presence of a compound with 754.3092 *m/z* at RT 12 min (Figure 2G) distinguishes the samples in the lower half of Figure 2D from the other samples. This finding is confirmed by extracted ion chromatograms (EICs) created for *m/z* 754.3092 (Figure 2F) and the box-and-whisker plot (Figure 2H). It is important to note that the multivariate analysis is used to identify important/discriminatory compounds/features within the dataset and the confirmation of their importance should always be achieved by extracting the representative data to ascertain the behaviour of these compounds across the sample set. The 754 ion was identified as rifamycin B (data not shown), a molecule reported in previous work by our group [35]. Identified compounds are listed in Table 1.

**Table 1:** Identification of six compounds from *S. arenicola* and *S. pacifica*.

Molecular Formula	Overall Score*	Mass^**^	Polarity	RT (min)	Difference (MFG, ppm)	Compound identification***	Example species
**C_37_H_45_NO_12_**	99.35	695.2937	Negative	21.36	0.68	Rifamycin S	*S. arenicola*
**C_22_H_37_NO_6_**	94.91	411.2628	Positive	8.12	3.13	Saliniketal B	*S. arenicola*
**C_22_H_37_NO_5_**	93.72	395.2661	Positive	10.61	2.72	Saliniketal A	*S. arenicola*
**C_39_H_49_NO_14_**	91.34	754.3092	Negative	12.20	0.52	Rifamycin B	*S. arenicola*
**C_23_H_31_N_3_O_3_**	79.43	397.2365	Positive	9.91	2.12	Cyclomarazine A	*S. arenicola*
**C_17_H_24_O_4_**	61.87	292.1675	Positive	7.87	3.45	Salinipyrone A	*S. pacifica*

The proposed formula obtained after the PCA and OPLS-DA analysis according to high-resolution LC-QTOF-MS measurements.

*Overall sc°re calculated from the empirical formula match with the database search. ^**^Neutral mass calculated for each compound.

^***^Compound identification performed through the in-house accurate mass database match, MS/MS fragmentation and reference standards.

The accurate mass *m/z* values from high resolution measurements highlighted by PCA are used to generate molecular formulae in order to propose putative compounds. As molecular weight increases so does the number of possible molecular formulae [36]. Compound proposals retained after both statistical and visual/manual curation were compiled to a list of accurate mass values, corresponding putative molecular formulae, RTs and IDs (Table S2), and this list was used for future targeted analysis of the 46 strains.

**Figure 3:**
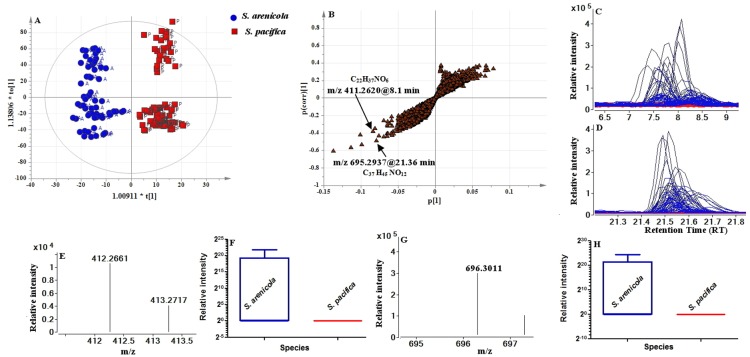
Metabolic profiling distinguishes bacterial samples from two *Salinispora* species *S. arenicola* and *S. pacifica*. (**A**) OPLS-DA scores plot, predictive component t[1] versus orthogonal component (t_0_ [1]) showing the supervised separation between the two sample classes. The ellipse in A and B represents the Hotelling's T^2^ 95% confidence interval for the multivariate data. (**B**) Loading S-plots derived from the LC-MS data set of two bacterial species. S-plot shows predictive component p[1] against the correlation p (corr) of variables of the discriminating component of the OPLS-DA model. (**C**) EIC of *m/z* 411 from all samples. (**D**) EIC of *m/z* 695 from two bacterial species. (**E**) Mass spectrum for *m/z* 411. (**F**) Box-and-whisker plot for *m/z* 411 – significantly present in *S. arenicola* but absent from *S. pacifica* (P<.0001) (**G**) Mass spectrum for *m/z* 695. (**H**) Box-and-whisker plot for *m/z* 695 – significantly present in *S. arenicola* but absent from *S. pacifica* (P<.0001).

Supervised multivariate analysis methods such as OPLS-DA were used in order to retrieve the variables explaining the differences between the two species only, delineating this information from any interfering or confounding sources of variance. The scores plot Figure 3A from such an analysis shows a complete separation in the predictive (horizontal) component (R2X(cum) = 21.2%, R2Y(cum) = 92.0%, Q2(cum) = 45.6%) which simplifies the interpretation of which variables contribute to a distinct species-specific difference. By this analysis it can be seen that there can indeed be a distinction made between species based on their chemotypes. Variation observed in the orthogonal (vertical) component is unrelated to strain differences but may warrant further investigation given that there are different strains of each species.

The S-plot visualizes the correlation and model influence of the metabolites (variables). As shown in Figure 3B, the variables to the extreme of the lower left quadrant are influential, with good reliability (correlation) and the highest (absolute) loadings scores. In the comparison of two groups, *S. arenicola* versus *S. pacifica*, the *S. pacifica* metabolites are comparatively more abundant in the upper right quadrant (with >0 correlation and model influence i.e. loadings), whereas in the lower left quadrant (with <0 correlation and model influence) these metabolites are more abundant in *S. arenicola*. Figures 3C–3H show the EICs, mass spectra and box-and-whisker plots used in the identification of rifamycin S and saliniketal B. From the interrogation of the OPLS-DA loadings plot, S-plot and Variable Importance in Projection (VIP) results, 57 metabolites were listed according to their highest VIP scores Table S2. To further investigate the significance of changes in these metabolites, a combination of approaches was used: t-test, VIP values and EICs, and finally results were combined with PCA results (Table S2). Analysis by the most complex methods used in this section clearly shows a distinction between *Salinispora* species on the basis of large scale secondary metabolite production.

### Identification of rifamycin O and W

In order to identify a previously unknown source of rifamycin O and W, an integrated approach was adopted consisting of the following steps: (1) HPLC-QToF-MS analysis with multimode ionisation (ESI and APCI) and fast polarity switching; (2) database searching of monoisotopic masses (tolerance 5 ppm); (3) matching of monoisotopic masses, retention times, mass spectra of molecular and fragment ions of the postulated compound and the authentic reference standards. Rifamycin W was not commercially available and was therefore tentatively identified based on structure using the database matching of the monoisotopic mass in conjunction with the mass spectrum of the molecular and fragment ion.

As shown in Figure S1, LC-UV-Vis (at 430 nm) chromatograms of *S. arenicola* strains MV0318 and MV0472, as well as M413 (ACM 5232) showed two peaks that eluted at times that were different to those found in our previous studies of rifamycins B, S and SV [35]. The bacterial strains previously studied included M403, SW15, M102, M413, SW10, SW17, M414, M412, SW 02 and M101. These two peaks were absent in the chromatograms of blank extracts produced using the sterile culture medium, and in chromatograms of rifamycin standards B and SV run using the same gradients as in this study. Therefore these peaks appear to be specific to the strains analysed in this study and as such are previously undetected metabolites.

**Figure 4:**
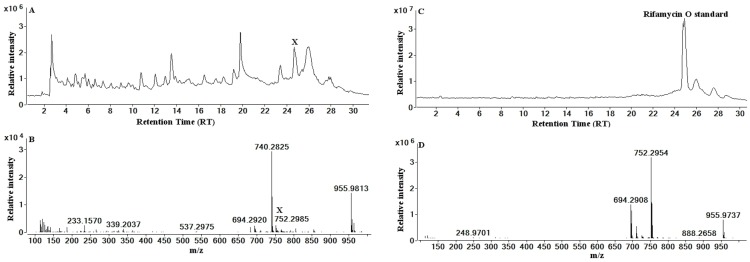
Chromatograms and mass spectra relating to the detection of rifamycin O. (**A**) LC-QToF-MS Total Ion Chromatogram (TIC) for *S. arenicola* strain MV0472 and (**B**) Mass spectrum of peak X and (**C**) Chromatogram for rifamycin O standard and (**D**) Mass spectrum of peak X (lower 2 panels). The retention times of X and rifamycin O standard are 24.5 and 24.55 min, respectively. The *m/z* of molecular ion of X and rifamycin O standards are 752.2985 and 752.2954.

The unknown peak eluting at 24.5 min was identified on the basis of its accurate mass, through an in-house database search, as rifamycin O; the monoisotopic mass of the unknown was 752.2985 [M-H]^−^ and that of rifamycin O in the database was 753.2996 [M]. The negative ionisation mode LC-MS chromatograms and the mass spectra of the peaks of interest for the extracts and rifamycin O standard are shown in Figure 4. Matching of the retention times (24.5 for standard and 24.55 for unknown) and the mass spectra further strengthened the identification through the database search. As described in the experimental section, chromatograms were obtained for both the extract and the standard rifamycin O, using the detection of peaks by MS/MS fragmentation spectra in negative ionisation mode. Figure S2 shows that the observed spectra and the proposed fragmentation pathways for the unknown peak and that of rifamycin O are the same, confirming the identity of the unknown peak to be rifamycin O. Figure S3 demonstrates the fragmentation of the rifamycin molecule to produce the major fragments observed in Figure S2 (*m/z* 694, 636, 514, 453 and 272).

In MS/MS mode, 752.2985 [M-H]^−^ was selected as the precursor ion. Both unknown and standard peaks produced fragment ions with identical *m/z* values. The presence of *m/z* 272 (naphthofuran) provides strong evidence for the identification of rifamycin [35]. It is worth mentioning the unique strength of LC-QToF-MS/MS technology in confirming the identity; we were able to detect the unknown peak with only 3 ppm mass difference from the standard rifamycin O, and in MS/MS mode the differences between standard and unknown spectra for 752, 694 and 272 ions were 0, 0 and 0.1 ppm, respectively. The confirmation of the identity in this case was based on several different observations: absorbance at 430 nm; matching the monoisotopic mass with an in-house database entry for rifamycin O; matching of three properties (retention time, mass spectra and fragmentation spectra) with standard rifamycin O; and the likely presence of a naphthofuran system in the fragmentation spectrum.

**Figure 5:**
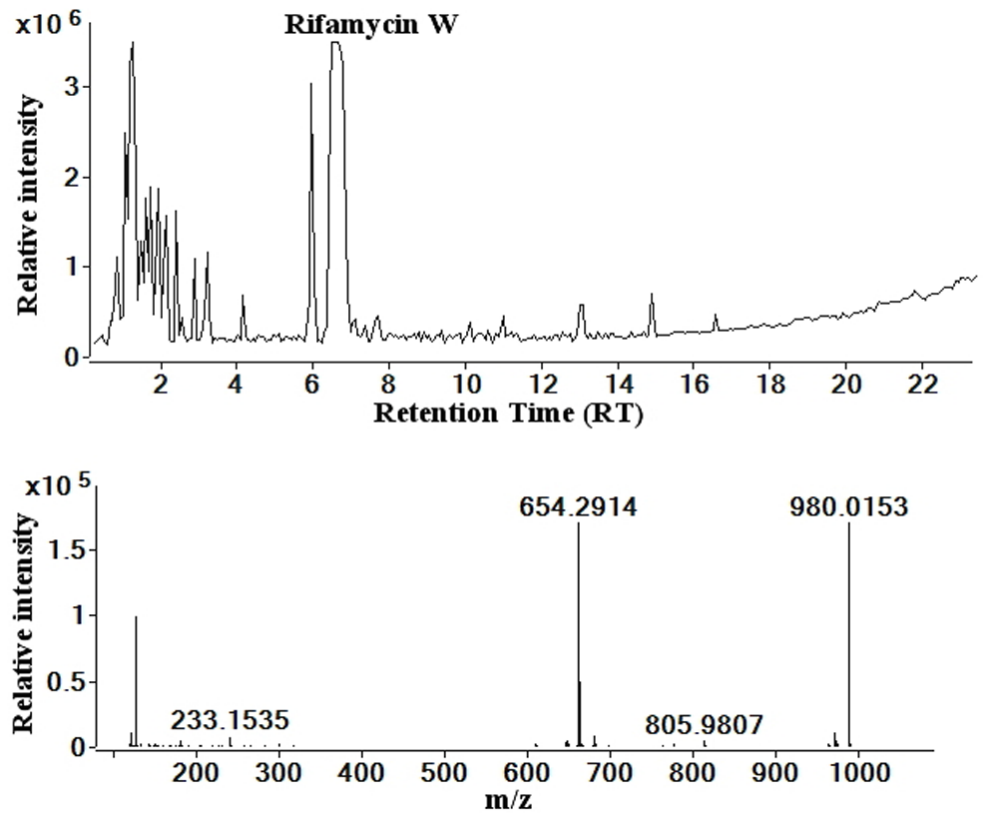
Total Ion Chromatogram for *S. arenicola* strain M413 (ACM5232) and the mass spectrum for peak Y.

The unknown peak eluting at 7 min was identified through an in-house database search as rifamycin W; the monoisotopic mass of the unknown was 654.2914 [M-H]^−^ (Figure 5) and that of rifamycin W in the database was 655.2992 (5 ppm tolerance; *m/z* 654.2914 [M-H]^−^). Figure 5 shows the negative ionisation LC-MS chromatogram and mass spectrum for the above unknown peak for *S. arenicola* extract of strain M413 (ACM5232). As rifamycin W is not commercially available, it was not possible to obtain the same data for an authentic standard. Following a similar protocol to the rifamycin O above, MS-MS fragmentation spectra for the unknown peak were obtained in negative ionisation mode, as shown in Figure S4. Four major fragments 452, 330, 272 and 245 have been identified and the generation of these product ions from the precursor ion is demonstrated in Figure S5. The confirmation of the identification of the unknown peak as rifamycin W in this case was based on several different observations: absorbance at 430 nm; matching of the mass obtained for the molecular ion in mass spectrum to that in database entry for rifamycin W (0.78 ppm difference); matching the product ions obtained in MS-MS spectrum from rifamycin W with fragmentation patterns; and the presence of the naphthofuran system in the fragmentation spectrum.

## Discussion

Analysis of the inter-species level of chemical diversity is important, not only in the context of speciation but also for understanding genetic variation during adaptive evolution within species, and to exploit the full diversity of natural products which may be available for biopharmaceutical screening and dereplication [37]. To date, most of the studies based on *Salinispora* species have focused on a limited number of targeted compounds rather than on chemical or metabolic traits with known functional roles. However, knowledge of the broader scale of secondary metabolite production in this obligate marine actinobacterium is of considerable interest in ecology and evolutionary biology as well as pre-filtering of strains during screening programs for the most likely producers of new biopharmaceuticals. ‘Omics’ approaches have thus far proven useful in providing some insight when attempting to answer complex biological questions of a similar nature [38], but more direct metabolomic and phenotypic approaches may also prove productive. Here, we have applied a mass spectrometry-based metabolomics approach to attempt to discover the concealed secondary metabolome in two *Salinispora* species: *S. arenicola* and *S. pacifica*. Our results show that screening a large number of compounds in bacterial species can answer questions relating to which metabolites are produced. This could be a key to answering the question of what their role may be in the adaptation of the organism to its environment and the effects on their immediate community.

With this work we have begun to document the chemical diversity in two *Salinispora* species collected from the Great Barrier Reef off, situated of the north east coast of Australia, an area extending over ∼2500 km. There are also similarities in the species-related chemotypes, which indicates the presence of common compounds in these two bacterial species. To remove confounding variation in the dataset and to focus the analysis on the factor of interest, namely species difference, supervised multivariate analysis highlighted that clear chemotypic differences exist between the two bacterial species, as detailed in Table S2. Interestingly, we have found rifamycins and saliniketal A and B to be consistently present of in all of the *S. arenicola* samples. Although we have identified rifamycins and saliniketals in *S. arenicola* species, we were unable to detect any specific compound class as present in *S. pacifica* as a whole. However, the ability of *S. arenicola* to synthesize rifamycins and saliniketals seems to form a definite species-specific character distinguishing *S. arenicola* from *S. pacifica*.

The detection of two rifamycin compounds among strains of *S. arenicola* was surprising given that these compounds had previously been observed exclusively in the terrestrial soil actinobacterium *Amycolatopsis mediterranei* [21], [39]. Out of 36 *Salinispora* strains screened in this study, only two strains produced detectable concentrations of rifamycin O, and one produced rifamycin W, as would be expected if a biosynthetic pathway had been inherited from *Amycolatopsis mediterranei* [20]. Nevertheless, most strains showed three peaks at retention times corresponding to previous work relating to other rifamycins (S, SV and B) [35]. Wilson and co-workers have reported that different strains of *Amycolatopsis mediterranei* produce different rifamycins [39]. Moreover, these rifamycins are subject to inter-conversion as a result of their physicochemical properties and vary considerably in their antimicrobial spectrum and the extent of their biological activities [40]; for example, the 16,17,18,19-tetrahydro analogue of rifamycin SV is three-fold less potent than rifamycin SV against *E. coli* polymerase [23]. It has been reported that rifamycin W is the precursor for rifamycin S, SV, B, L and Y [22], [39]. In previous studies we found that rifamycin B production is higher in all the strains and is converted to rifamycin SV and S over time [35]. Similarly, Banerjee and colleagues found that rifamycin B is a precursor for rifamycin Y and other rifamycin analogues [24]. The presence of the enzyme rifamycin oxidase facilitates the biotransformation process from rifamycin B to rifamycin S, a stable analogue with good antimicrobial activity.

There is a high similarity between the *rif* gene sequence found in *Amycolatopsis mediterranei* and *Salinispora* sp. (99% as assessed by neighbour-joining bootstrap values)and the study suggests that the gene has been exchanged horizontally [9]. However, it is not clear at what point during the evolution of the *Salinispora* genus that the pathway may have been acquired. The rifamycin O and W genes were not found to be present in the *S. arenicola* genome from a previous study by Wilson and co-workers [39]. In our study, the presence of rifamycins O and W in the *S. arenicola* secondary metabolome at least in some strains suggests that *rif* O and W genes were acquired by HGT due to the selective advantage these antimicrobial compounds confer in their environment. This evolutionary history is what might be expected if the acquisition of pathways facilitates ecological diversification or a selective sweep [41] resulting from strong selection for the acquired pathway, either of which provide compelling evidence that the production of secondary metabolites has great impact on creating functional traits with important ecological roles. The concept that gene acquisition provides a mechanism for ecological diversification that may finally drive the formation of independent bacterial lineages has been previously proposed [42]. Interestingly the inclusion of secondary metabolism among the functional categories of acquired genes that may have this effect shows the importance of the functional and evolutionary significance of these gene clusters [26]. Inferring the evolutionary histories of the biosynthetic pathways associated with secondary metabolism remains complex but provides a possibility to understand how nature creates structural diversity and the extent to which this diversity is related to phylogenetic grouping.

A previous study of the marine actinobacteria *Salinispora* showed that this genus produces secondary metabolite profiles that are species-specific, for instance Jensen and co-workers have reported core compounds that are identical in each species [15]. To date, traditional screening techniques, for instance bioactivity guided assays [43], and subsequently genome mining techniques [27] have mainly been used to isolate and identify secondary metabolite profiles of *Salinispora*. However, these studies have been constrained to targeted compounds and have not focused on a broader investigation of the wider metabolome. Our results show that the application of UHPLC-QToF-MS and chemometric approaches can be used to successfully differentiate and discriminate between two species and possibly identify new compounds. In this instance the goal of the metabolomic profiling was to discover new biochemical descriptors and was essentially a semi-quantitative analysis. However, these data lend themselves to directed interrogation if specific compounds (e.g. rifamycins) are also of interest, therefore providing a “best-of-both-worlds” outcome. Targetted MS/MS experiments were performed when specific results were required (e.g. rifamycins O and W).

Despite sample quantity and availability being an issue in marine natural product research we found distinct metabolite profiles to be present in each species with some degree of commonality between the two. These findings extend those of Jensen (2007), confirming that species-specific metabolites are produced by *S. arenicola* and *S. pacifica*. The combination of MS-based metabolic profiling and chemometrics enables the discovery and identification of a larger proportion of the secondary metabolome than available with traditional approaches. However, the cross-over between studies is incomplete and this could be due to a number of factors, not least the variability of culture medium conditions [44] as well as geographical differences (even in centimetre scale collections) [26], which undoubtedly play important roles in secondary metabolite production. However, this study provides compelling evidence that a metabolomic profiling approach affords an efficient and effective tool for natural product discovery. Consequently, the distinctions in profiles noted in our study were unrelated to a specific biological activity or aimed at only targeted compounds. This study therefore indicates the benefit of using high resolution accurate mass spectrometry and chemometric analysis in exploring microbial metabolite profiles, as it is rapid and reproducible, and only a small amount of experimental sample is required to obtain valuable information. Most notably, this is the first study to our knowledge to investigate the feasibility of using UHPLC-QToF-MS and chemometrics to explore metabolite profiles in the marine natural product-synthesizing actinobacterium *Salinispora*.

In conclusion, we have shown that the diversity of two *Salinispora* species, based on their metabolomes and thus natural product chemotypes, is significantly greater than suggested by previously identified compounds. Our data reveals that the qualitative variation in the (bio)chemical profiles of the two species provides a major source of differentiation between these species, in addition to previous genetic and targeted chemical classification. We can now appreciate the spectrum of secondary metabolomes in *Salinispora* strains as much wider than already described compounds from these species, providing a rainbow of new natural product ‘colours’ for biodiscovery from these important marine bacteria.

## Supporting Information

Figure S1 **LC-UV-Vis (430 nm) chromatograms of** ***S. arenicola*** **strains (A) MV0472, (B) MV0318, and (C) M413 (ACM5232) showing 2 new unknown peaks X and Y.**
(TIF)

Figure S2 **Negative mode MS/MS fragmentation spectra (A) Rifamycin O standard (B) Peak X.** Major fragments observed were *m/z* 694.2872, 636.2470, 514.1703, 392.1159 and 272.0554 for X and *m/z* 694.2872, 636.2740, 514.1755, 392.1137 and 272.0555 for rifamycin O standard.
(TIF)

Figure S3 **Proposed MS/MS fragmentation pattern of rifamycin O in negative ionization mode.**
(TIF)

Figure S4 **Negative mode MS/MS fragmentation of peak Y.** Major fragments are *m/z* 624.2823, 538.2089, 452.1729, 330.0993, 272.0567 and 245.0333.
(TIF)

Figure S5 **Proposed MS/MS negative mode fragmentation pattern of rifamycin W.**
(TIF)

Figure S6 **Full total ion current (TIC) chromatograms of representative extracts from** ***S. arenicola*** **and** ***S. pacifica*** **.**
(TIFF)

Table S1 **QToF-MS operational conditions (switching).**
(DOCX)

Table S2 **Empirical formula generated from 46 strains of** ***S. arenicola*** **and** ***S. pacifica*** **from Great Barrier Reef (GBR) regions.** The proposed formula obtained after the PCA and OPLS-DA analysis according to high-resolution LC-QToF-MS measurements.
(DOCX)

Text S1 (DOCX)
