# Supplementary figures and images for: Discovering the Recondite Secondary Metabolome Spectrum of Salinispora Species: A Study of Inter-Species Diversity
Source: PLoS One. 2014 Mar 12;9(3):e91488. doi: 10.1371/journal.pone.0091488 (PMC3951395; doi:10.1371/journal.pone.0091488)

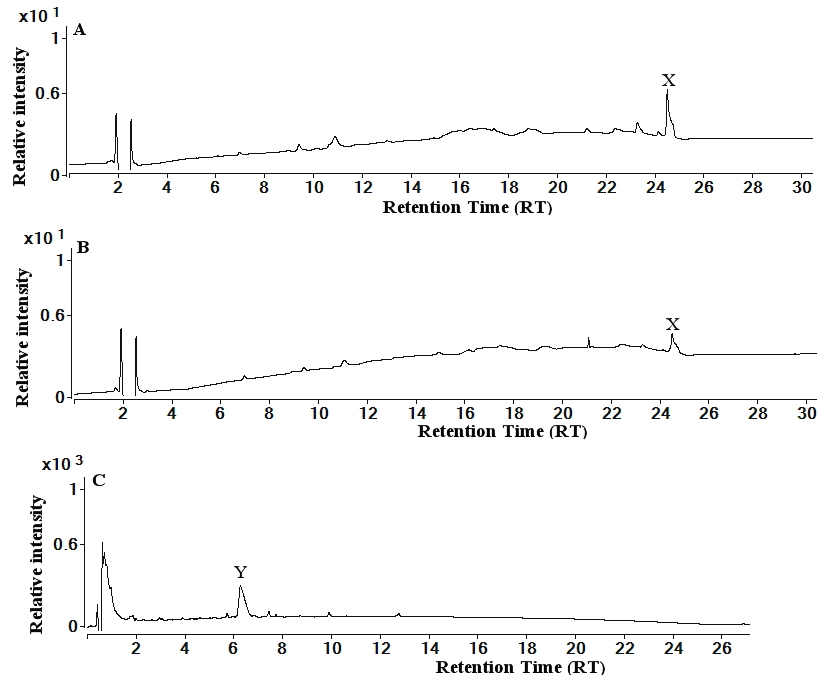

Supplement: Figure S1 — LC-UV-Vis (430 nm) chromatograms of S. arenicola strains (A) MV0472, (B) MV0318, and (C) M413 (ACM5232) showing 2 new unknown peaks X and Y. (TIF) [file pone.0091488.s001.tif]

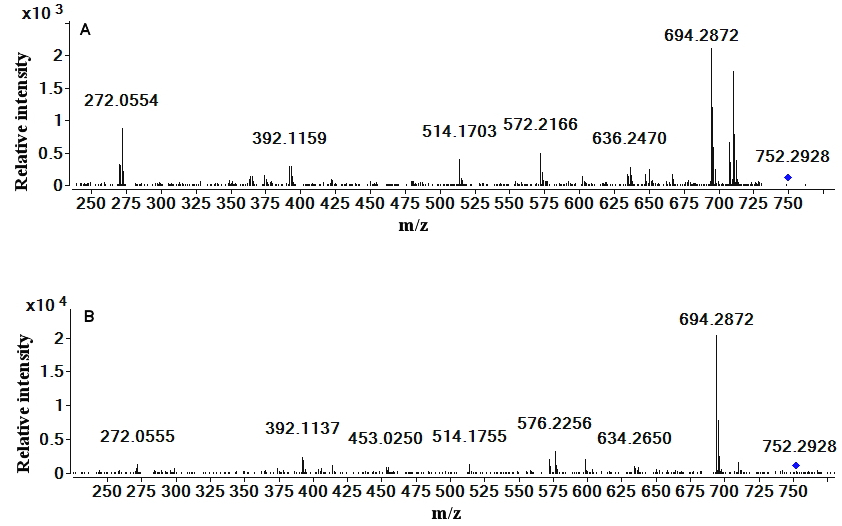

Supplement: Figure S2 — Negative mode MS/MS fragmentation spectra (A) Rifamycin O standard (B) Peak X. Major fragments observed were m/z 694.2872, 636.2470, 514.1703, 392.1159 and 272.0554 for X and m/z 694.2872, 636.2740, 514.1755, 392.1137 and 272.0555 for rifamycin O standard. (TIF) [file pone.0091488.s002.tif]

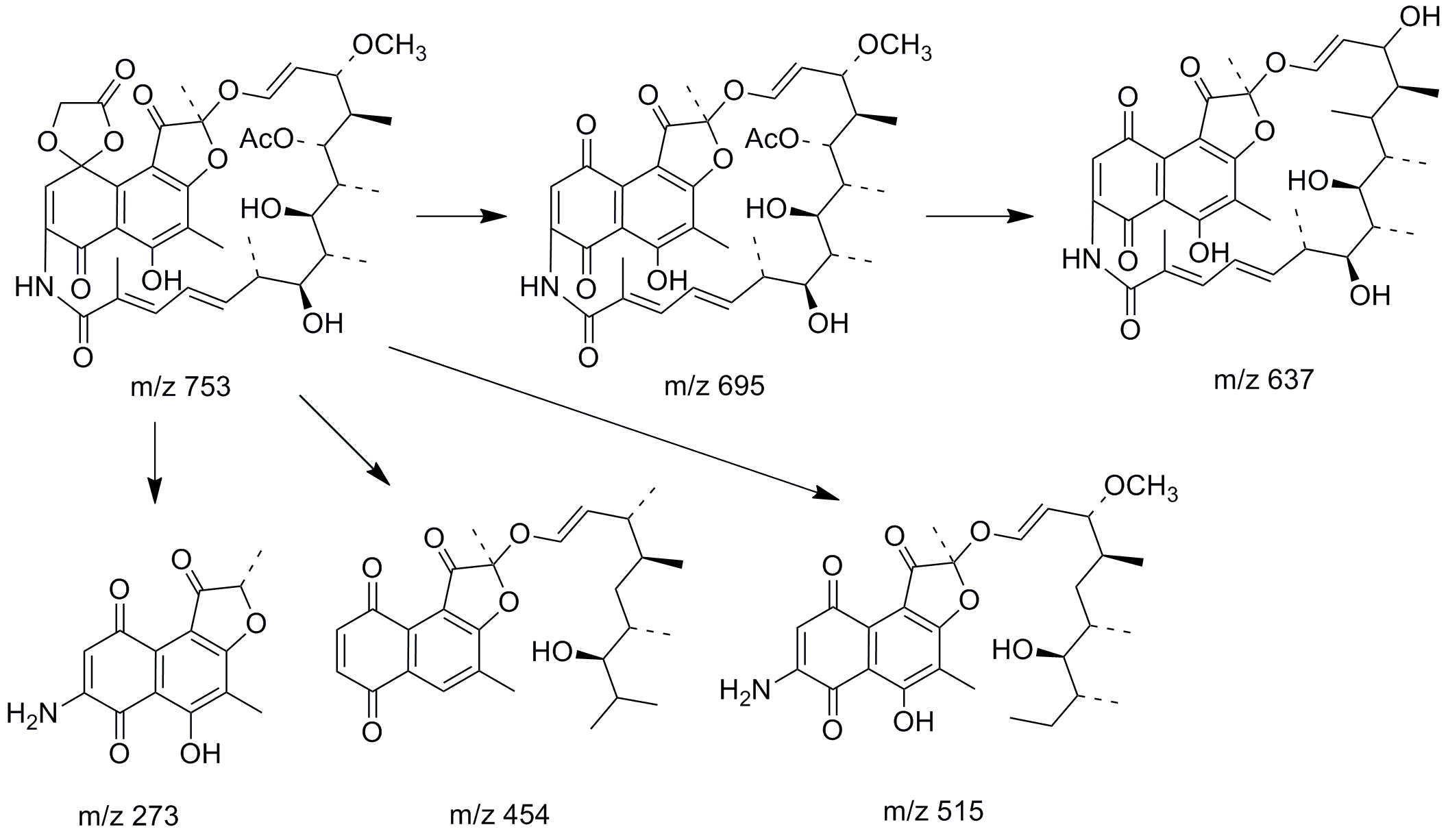

Supplement: Figure S3 — Proposed MS/MS fragmentation pattern of rifamycin O in negative ionization mode. (TIF) [file pone.0091488.s003.tif]

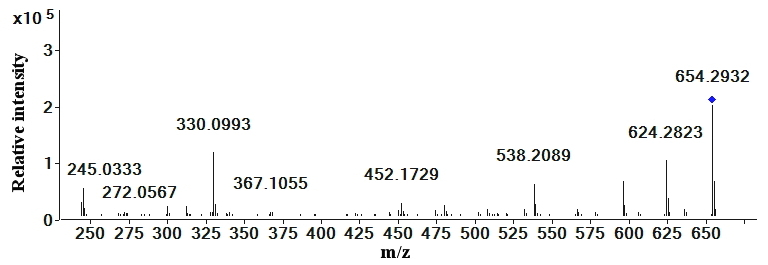

Supplement: Figure S4 — Negative mode MS/MS fragmentation of peak Y. Major fragments are m/z 624.2823, 538.2089, 452.1729, 330.0993, 272.0567 and 245.0333. (TIF) [file pone.0091488.s004.tif]

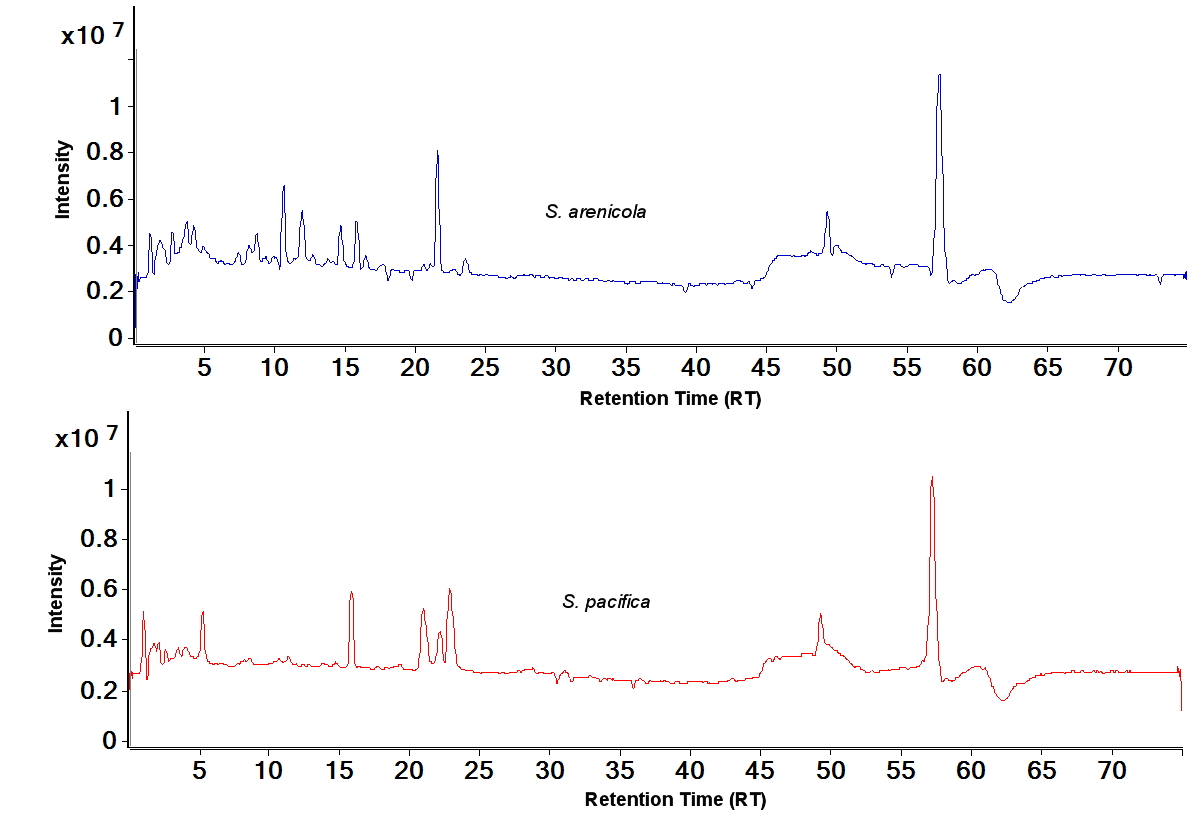

Supplement: Figure S6 — Full total ion current (TIC) chromatograms of representative extracts from S. arenicola and S. pacifica . (TIFF) [file pone.0091488.s006.tiff]
