# Supplementary material for: Discovering the Recondite Secondary Metabolome Spectrum of Salinispora Species: A Study of Inter-Species Diversity
Source: PLoS One. 2014 Mar 12;9(3):e91488. doi: 10.1371/journal.pone.0091488 (PMC3951395; doi:10.1371/journal.pone.0091488)
Supplement: Table S1 — QToF-MS operational conditions (switching). (DOCX) [file pone.0091488.s007.docx]

**Table S1**: QToF-MS operational conditions (switching)

**Parameters**

Capillary voltage (VCap) 2500

Nebulizer pressure (psig) 30

Drying gas (L/min) 5.0

Gas temperature (ºC) 300

Vaporizer 200

Voltage charge (VCharge) 2000

Mass range (m/z) 100-1000

**Scan Segments**

Scan Seg Ion Polarity

1 Negative

2 Positive

**Scan segment 1**

Scan source parameters

Parameter Value

VCap 2500

Corona Negative 15.0

Fragmentor 175

Skimmer1 65.0

OctopoleRFPeak 750

**Scan segment 2**

Scan source parameters

Parameter Value

VCap 2500

Corona Positive 4.0

Fragmentor 175

Skimmer 1 65.0

OctopoleRF Peak 750
