# Supplementary material for: Discovering the Recondite Secondary Metabolome Spectrum of Salinispora Species: A Study of Inter-Species Diversity
Source: PLoS One. 2014 Mar 12;9(3):e91488. doi: 10.1371/journal.pone.0091488 (PMC3951395; doi:10.1371/journal.pone.0091488)
Supplement: Table S2 — Empirical formula generated from 46 strains of S. arenicola and S. pacifica from Great Barrier Reef (GBR) regions. The proposed formula obtained after the PCA and OPLS-DA analysis according to high-resolution LC-QToF-MS measurements. (DOCX) [file pone.0091488.s008.docx]

**Table S2:** Empirical formula generated from 46 strains of *S. arenicola* and *S. pacifica* from Great Barrier Reef (GBR) regions. The proposed formula obtained after the PCA and OPLS-DA analysis according to high-resolution LC-QToF-MS measurements.

| Molecular Formula | Overall  Score^*^ | Mass^**^ | Polarity | RT (min) | Difference  (MFG, ppm) |
| --- | --- | --- | --- | --- | --- |
| C_11_H_18_N_2_O_3_ | 99.99 | 226.1317 | Positive | 1.83 | 0.18 |
| C_24_H_36_O_5_ | 97.38 | 404.2563 | Positive | 8.22 | -1.72 |
| C_22_H_26_O_6­_ | 95.23 | 386.1712 | Positive | 16.35 | 7.97 |
| C_11_H_18_N_2_O_2_ | 95.02 | 210.1358 | Positive | 2.29 | 4.89 |
| C_26­_H_41_NO_4_ | 94.74 | 431.3028 | Positive | 10.64 | 1.66 |
| C_11_H_18_N_2_O_3_ | 94.04 | 226.1328 | Positive | 1.78 | -4.6 |
| C_20_H_32_O_5_ | 93.42 | 352.224 | Positive | 5.12 | -1.05 |
| C_24_H_38_O_4_ | 93.34 | 390.2783 | Positive | 48.02 | 0.11 |
| C_14_H_13_N_3_O | 90.41 | 239.1048 | Positive | 7.22 | 4.43 |
| C_20_H_28_O_4_ | 90.17 | 332.1975 | Positive | 3.01 | 7.82 |
| C_22_H_37_NO_5_ | 89.92 | 395.2658 | Positive | 10.96 | 3.47 |
| C_20_H_28_O_4_ | 88.41 | 332.1988 | Positive | 2.73 | -4.68 |
| C_27_H_41_N_5_O_2_ | 87.76 | 467.3248 | Negative | 11.27 | -0.19 |
| C_22_H_37_NO_6_ | 87.27 | 411.2605 | Positive | 4.93 | 3.86 |
| C_25_H_37_N_5_O | 86.13 | 423.2978 | Positive | 7.94 | -1.64 |
| C_33_H_41_N_7_O_5_S_2_ | 85.44 | 679.2613 | Positive | 9.87 | 0.34 |
| C_16­_H_22_O_4_ | 85.21 | 278.1520 | Negative | 5.16 | 1.86 |
| C_25_H_37­_N_5_ | 84.60 | 407.3043 | Positive | 11.28 | -1.82 |
| C_8_H_6_N_2_O_2_ | 83.23 | 162.0425 | Negative | 1.94 | 1.73 |
| C_20_H_30_O_4_ | 82.93 | 334.2143 | Positive | 3.78 | 2.38 |
| C_22_H_33_NO_5_ | 81.72 | 391.2336 | Positive | 9.38 | 2.38 |
| C_22_H_36_O_3_ | 81.59 | 348.2656 | Positive | 15.07 | -1.5 |
| C_24_H_38_O_4_ | 81.53 | 390.2270 | Negative | 12.53 | -1.0 |
| C_24_H_30_O_8_ | 81.27 | 446.1941 | Positive | 15.79 | 0.64 |
| C_26_H_42_O_6_ | 80.82 | 450.2986 | Negative | 11.72 | -1.02 |
| C_12_H_16_N_4_O_2_ | 78.47 | 248.1267 | Positive | 3.51 | -2.87 |
| C_28_H_42_O_7_ | 76.25 | 490.2919 | Positive | 2.13 | 1.11 |
| C_22_H_26_O_6_ | 75.79 | 386.1740 | Positive | 14.11 | -2.75 |
| C­_20_H_29_N_3_O_7_ | 75.70 | 423.2013 | Positive | 2.73 | -1.73 |
| C_26_H_41_NO_4_ | 75.35 | 431.3027 | Positive | 9.99 | 0.55 |
| C_24_H_38­_O_4_ | 75.32 | 390.2763 | Positive | 47.65 | 0.15 |
| C_20_H_31_NO_4_ | 74.82 | 349.2242 | Positive | 7.46 | -1.94 |
| C_22_H_36_O_3_ | 72.59 | 348.2665 | Positive | 14.05 | 3.04 |
| C_29_H_39_NO_5_ | 71.55 | 481.2860 | Positive | 11.31 | 0.32 |
| C_22_H_30_O_3_ | 69.68 | 342.2182 | Positive | 10.61 | 3.77 |
| C_18_H_25_N_3_O_6_ | 68.29 | 379.1743 | Positive | 3.32 | 0.29 |
| C_34_H_54_O_8_ | 67.70 | 590.3796 | Positive | 32.33 | 0.73 |
| C_36_H_65_NO_13_ | 66.28 | 719.4466 | Positive | 18.69 | 0.42 |
| C_22_H_37_NO_5_ | 66.22 | 395.2647 | Positive | 4.98 | -0.13 |
| C_17_H_27_BrO_2_ | 64.75 | 642.1191 | Positive | 1.87 | -1.98 |
| C_22_H_33_NO_5_ | 63.28 | 391.2347 | Positive | 7.15 | -1.54 |
| C_28_H_35_NO_6_ | 63.03 | 481.2510 | Positive | 9.62 | 0.01 |
| C_20_H_34_N_2_O_6_ | 62.06 | 398.2417 | Positive | 26.54 | 1.69 |
| C_29_H_44_O_6_ | 60.95 | 488.3136 | Negative | 2.12 | -1.46 |
| C_22_H_37_NO_6_ | 60.91 | 411.2600 | Positive | 6.27 | -1.01 |
| C_29_H_39_NO_5_ | 60.14 | 481.2828 | Positive | 10.75 | -0.43 |
| C_11_H_14_O_2_ | 59.53 | 178.0994 | Positive | 3.33 | -3.48 |
| C_25_H_49_ClN_6_O_8_ | 58.78 | 596.3314 | Positive | 25.38 | 0.57 |
| C_20_H_30_O_4_ | 58.74 | 334.2131 | Positive | 4.26 | 3.92 |
| C_20_H_26_O_3_ | 57.80 | 314.1868 | Positive | 2.74 | 4.44 |
| C_24_H_32_O_3_ | 57.53 | 368.2351 | Positive | 8.21 | 3.23 |
| C_22_H_32_O_4_ | 56.91 | 360.2301 | Positive | 10.99 | 6.63 |
| C_32_H_50_O_8_ | 56.54 | 562.3479 | Positive | 26.88 | 0.71 |
| C_11_H_14_N_4_O_2_ | 56.07 | 234.1117 | Positive | 1.52 | 0.09 |
| C_20_H_28_O_3_ | 55.92 | 316.2055 | Positive | 8.127 | -5.23 |
| C_21_H_28_O_8_ | 53.10 | 408.1755 | Positive | 1.56 | -0.56 |
| C_20_H_26_O_3_ | 52.34 | 314.1869 | Positive | 3.56 | 4.12 |
