# Supplementary material for: Discovering the Recondite Secondary Metabolome Spectrum of Salinispora Species: A Study of Inter-Species Diversity
Source: PLoS One. 2014 Mar 12;9(3):e91488. doi: 10.1371/journal.pone.0091488 (PMC3951395; doi:10.1371/journal.pone.0091488)
Supplement: Text S1 — (DOCX) [file pone.0091488.s009.docx]

Text S1

**Cultivation of strains and metabolite production for rifamycin W**

For 22 strains, including MV0318 and MV0472, the time from cultivation to harvesting and extraction was 56 days and for the remainder, including M413 (ACM5232), it was 14 days.

**HPLC-MS analysis**

Chromatographic separations were performed using an Agilent 1290 HPLC system (Agilent Technologies, Santa Clara, USA), and the detection was accomplished via a serially connected Agilent 1290 Infinity photodiode array detector and an Agilent 6520 accurate mass QToF mass spectrometer equipped with a multimode source (Agilent). Separations were achieved using an Agilent Eclipse ZORBAX Plus C18 column (2.1×150mm, 3.5µm; Agilent, USA) with a ZORBAX Reliance cartridge guard column (4.6 X 12.5mm, 5µm) for the rifamycin O study and an Agilent ZORBAX Eclipse Plus C18 (2.1 x 50 mm, 1.8 µm) HPLC column for the rifamycin W study. Experimental UV-DAD and MS data were acquired by MassHunter acquisition software version B. 02.01 SP3 (Agilent).

The chromatography was achieved using a gradient elution with following mobile phases: 100% water (mobile phase A) and 100% acetonitrile (mobile phase B) at a flow rate of 0.2 mL min^-1^. For the rifamycin O study, the gradient used was as follows: 20% to 55% B for first 20 min, 55% to 100% B from 20 to 23 min and held at 100%B from 23 to 30 min. The composition of the eluent was then restored to 20%B within 3 min and the system was re-equilibrated for 10 min. For the rifamycin W study, the gradient used was as follows: 20% to 100% B for the first 25 mins, maintained at 100% B for 2.5 mins and return to 20% B during the next 5 mins followed by equilibration at 20%B for 10 min.

The UV-Vis detector was set to detect signals at six different wavelengths including 430nm. A dual nebulizer electrospray source was used for continuous introduction of reference ions. In MS mode the instrument was operated in full scan mode from m/z 100 to 1000 for all samples at a scan rate of 0.8 cycles/s. This mass range enabled the inclusion of two reference compounds, a lock mass solution including purine (C_5_H_4_N_4_ at m/z 121.050873, 10 µm) and hexakis-(1H,1H,3H-tetrafluoro-pentoxy) phosphazene (C_18_H_18_O_6_N_3_P_3_F_24_ at m/z 922.009798, 2 µm). Multimode (Electrospray Ionisation (ESI) and Atmospheric Pressure Chemical Ionization (APCI)) with Fast Polarity Switching (FPS) was used to ionize and detect compounds after chromatographic separation. The general parameters of the MS1 mixed mode source were as follows: capillary voltage 2500 V, nebulizer pressure 30 psig, drying gas 5.0 l/min, gas temperature 300 ºC, vaporizer 200 V, voltage charge 2000 V; negative-ion mode capillary voltage 2500 V, corona negative 15.0 V, fragmentor 175 V, skimmer1 65.0 V, octopole RF Peak 750 V; positive ion mode capillary voltage 2500 V, corona positive 4.0 V, fragmentor 175V, skimmer1 65.0 V and octopole RF Peak 750 V.

MS/MS experiments were run in negative ionisation mode. The same MS1 gradient settings were used for MS/MS experiments. MS/MS conditions were as follows: source parameters- maximum precursor per cycle was 3, gas temp. 300 ̊C, vaporizer 200 V, gas flow (l/min) 10.0, nebulizer (psi) 30, VCharge 2000 V; scan source parameters: VCap 2500 V, corona negative 15.0 V, fragmentor 135V, skimmer1 65.0 V, Octopole RF peak 750 V.

**Materials**

Rifamycin O was purchased from TOCRIS Bioscience, Canada (Local distributor: Sapphire Bioscience Pty, Ltd., Australia). Rifamycins SV and B were from Sigma (St. Louis, MO, USA). All solvents used were of HPLC grade and supplied by Sigma Aldrich (Castle Hill, Sydney, Australia).
